# Knockdown of astrocytic Grin2a aggravates β‐amyloid‐induced memory and cognitive deficits through regulating nerve growth factor

**DOI:** 10.1111/acel.13437

**Published:** 2021-07-22

**Authors:** Zunshu Du, Yizhi Song, Xinyue Chen, Wanning Zhang, Guitao Zhang, Hui Li, Lirong Chang, Yan Wu

**Affiliations:** ^1^ Beijing Key Laboratory of Neural Regeneration and Repair Department of Anatomy School of Basic Medical Sciences Beijing Institute of Brain Disorders Capital Medical University Beijing China

**Keywords:** Alzheimer's disease, astrocyte, cognitive deficit, GluN2A, nerve growth factor, β‐amyloid

## Abstract

Synapse degeneration correlates strongly with cognitive impairments in Alzheimer's disease (AD) patients. Soluble Amyloid‐beta (Aβ) oligomers are thought as the major trigger of synaptic malfunctions. Our earlier studies have demonstrated that Aβ oligomers interfere with synaptic function through N‐methyl‐D‐aspartate receptors (NMDARs). Our recent in vitro study found the neuroprotective role of astrocytic GluN2A in the promotion of synapse survival and identified nerve growth factor (NGF) derived from astrocytes, as a likely mediator of astrocytic GluN2A buffering against Aβ synaptotoxicity. Our present in vivo study focused on exploring the precise mechanism of astrocytic GluN2A influencing Aβ synaptotoxicity through regulating NGF. We generated an adeno‐associated virus (AAV) expressing an astrocytic promoter (GfaABC1D) shRNA targeted to Grin2a (the gene encoding GluN2A) to perform astrocyte‐specific Grin2a knockdown in the hippocampal dentate gyrus, after 3 weeks of virus vector expression, Aβ were bilaterally injected into the intracerebral ventricle. Our results showed that astrocyte‐specific knockdown of Grin2a and Aβ application both significantly impaired spatial memory and cognition, which associated with the reduced synaptic proteins PSD95, synaptophysin and compensatory increased NGF. The reduced astrocytic GluN2A can counteract Aβ‐induced compensatory protective increase of NGF through regulating pNF‐κB, Furin and VAMP3, which modulating the synthesis, mature and secretion of NGF respectively. Our present data reveal, for the first time, a novel mechanism of astrocytic GluN2A in exerting protective effects on synapses at the early stage of Aβ exposure, which may contribute to establish new targets for AD prevention and early therapy.

## Graphical abstract

Using a Grin2a (the gene encoding GluN2A) shRNA adeno‐associated viral (AAV) vector, an astrocyte‐specific Grin2a knockdown rat AD model was established. The results showed astrocytic Grin2a knockdown aggravated Aβ‐induced memory and cognitive deficits through modulating the synthesis, mature and secretion of NGF. This in vivo data reveal, for the first time, a novel mechanism of astrocytic GluN2A in exerting protective effects on synapses at the early stage of Aβ exposure.

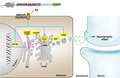

## 1 INTRODUCTION

Alzheimer's disease (AD) is characterized by progressive deterioration of memory and other cognitive functions. Synapse degeneration, as an early event in AD pathogenesis (Selkoe, 2002), correlates strongly with cognitive impairments in AD patients (Müller et al., 2017; Wang & Mandelkow, 2016). Emerging evidence implicates soluble Amyloid‐beta (Aβ) oligomers as the major trigger of synaptic malfunction and loss in AD patients brain.

Our own earlier studies and others evidence suggest that soluble Aβ oligomers interfere with synaptic function by acting as gain‐of‐function ligands that bind near, or to, ionotropic glutamate N‐methyl‐D‐aspartate receptors (NMDARs), which play a central role in mediating both physiological synaptic plasticity and glutamate‐induced neurotoxicity (Chang et al., 2016; Ferreira et al., 2015; Liu et al., 2010; Wang et al., 2013). In contrast to earlier views that NMDARs are exclusively expressed in neurons, recent two decades work demonstrates that fully functional NMDARs are present on astrocytes (Cahoy et al., 2008; Conti et al., 1996, 1999; Lee et al., 2010), although the precise function of astrocytic NMDARs remains enigmatic.

NMDARs, which are encoded by the Grin gene family, exist as hetero‐tetrameric complexes consisting of two obligatory GluN1 along with two GluN2(A‐D) and/or GluN3 (A‐B) subunits (Bai et al., 2013; Dumas, 2005; Myers et al., 2019). Our previous in vitro studies have found the neuroprotective role of astrocytic GluN2A subunit in the promotion of synapse survival, and identified nerve growth factor (NGF), a neurotrophin derived from astrocytes, as a likely mediator of astrocytic GluN2A buffering against Aβ synaptotoxicity (Li et al., 2016). NGF, as the first discovered and best characterized neurotrophin, its beneficial effects during early stage of AD have been verified (Guo & Mattson, 2000; Mattson, 2008; Schulte‐Herbruggen et al., 2007). NGF composes of α, β and γ three subunits, among them β subunit (β‐NGF) is responsible for its biological activity (Shooter, 2001; Young et al., 1988). Several researches have demonstrated that β‐NGF can protect synapses against Aβ‐induced neurotoxicity (Canu et al., 2017; Cuello et al., 2019). However, the exact nature of astrocytic GluN2A‐NGF interaction and the mechanisms involved in Aβ‐induced synaptotoxicity remain unclear. To clarify the related mechanisms of astrocytic GluN2A which protect neurons against the early Aβ synaptotoxicity through regulating NGF could provide a pharmacological strategies aiming to increase an endogenous neuroprotective NGF level for early treatment of AD.

The in vivo study reported here focused on whether and how astrocytic GluN2A subunit can influence the synaptotoxic effects of Aβ through regulating astrocytic NGF. Our results show that astrocyte‐specific knockdown of Grin2a (the gene encoding GluN2A) in the rat hippocampus can counteract Aβ‐induced compensatory protective increase of β‐NGF through modulating pNF‐κB, Furin and VAMP3, which affects the synthesis, mature and secretion of NGF respectively. Our in vivo findings reveal, for the first time, a novel mechanism of astrocytic GluN2A exerting protective effects on synapses at the early stage of exposure to Aβ oligomers.

## 2 RESULTS

### 2.1 Knockdown of astrocytic Grin2a aggravates Aβ‐induced memory and cognitive impairments

Our previous initial study has demonstrated that there were higher normal level and significant Aβ‐induced increase of astrocytic GluN2A in the neuron‐astrocyte mixed cultures compared to that in pure astrocytic cultures (Li et al., 2016), which implies that astrocytic GluN2A may play an enigmatic role in the complex cross talk of neuron‐astrocyte, including the neuronal response to Aβ. We also found that astrocytic GluN2A promotes synapse survival likely through NGF derived from astrocytes buffering against Aβ_1‐40_ synaptotoxicity (Li et al., 2016). In our recent in vitro experiments, with anti‐β‐NGF antibody to deplete β‐NGF in medium of neuron‐astrocyte co‐cultures, we have further confirmed astrocytic NMDA receptor protects neurons against Aβ_1‐42_‐induced synaptotoxicity through NGF secreted by astrocytes (data not shown). But the precise mechanism of astrocytic GluN2A in the execution of the synaptoprotective effects, especially in AD rat model, remains undefined.

**FIGURE 1:**
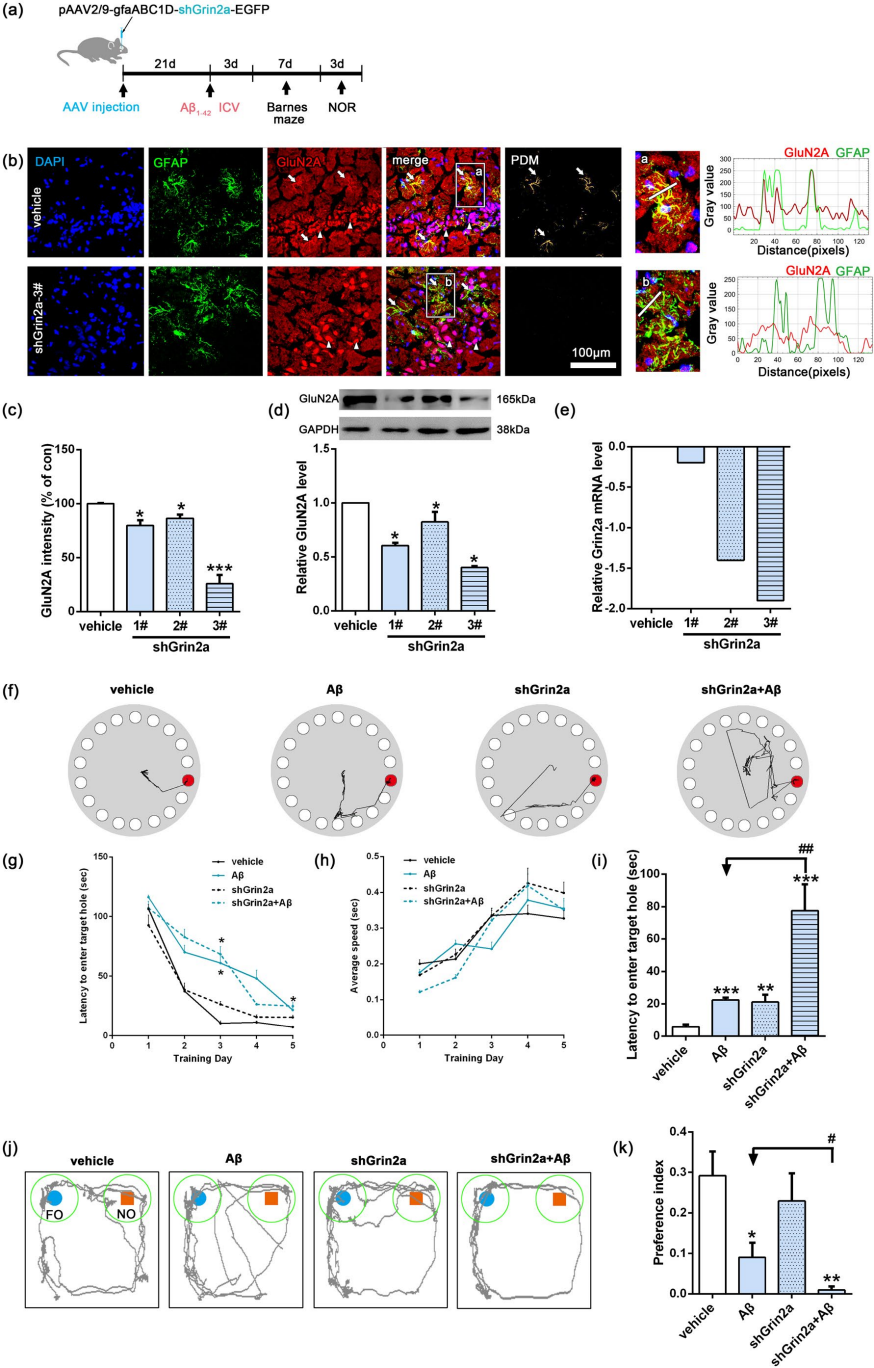
Knockdown of rat astrocytic Grin2a aggravates Aβ‐induced memory and cognitive impairments. (a) Schematic diagram of the experimental design. (b, c) Knockdown efficacy of astrocyte‐specific Grin2a was verified with confocal imaging. The fluorescence distribution along the linescan profile depicting the spatial distribution of GluN2A and GFAP. The subregion images (a and b, indicated by white rectangle on the left images) are magnified for clarity. White arrows indicating the GluN2A positive astrocytes, and white arrowhead indicating GluN2A positive neurons. Bar, 100 μm, *n* = 3 rats/group. (d) Representative western blot band and quantification showing the relative expression of GluN2A after AAVs injection. (e) The relative expression of GluN2A at mRNA level after AAVs injection. (f‐i) Barnes maze test showing astrocytic Grin2a knockdown aggravates Aβ‐induced memory and cognitive impairments. The representative locomotor traces of the four groups rats in the probe trial (f), latency to the target holes (g), average locomotor speed (h) during the training days and the latency to the target hole in the probe trial (i). (j, k) Novel objective recognition (NOR) test showing astrocytic Grin2a knockdown aggravates Aβ‐induced memory and cognitive impairments. The representative locomotor traces (j) and preference index (k) of the four groups rats. preference index is calculated as the time exploring the novel object divided by total time spent in exploring the familiar and the novel objects in the probe session. FO, familiar object; NO, novel object. Numerical data are shown as means ± SEM. *n* = 5–8 rats/group, * vs. vehicle, *p < 0.05, **p < 0.01, ***p < 0.01, ^#^ vs. Aβ, ^#^ *p* < 0.05, ^##^ *p* < 0.01

Our previous in vitro findings prompt us to further investigate the potential mechanism of astrocytic GluN2A in rat AD model. Initially, in order to reduce GluN2A expression in astrocytes of rat hippocampus, we established characterized Grin2a shRNA adeno‐associated viral (AAV) vector equipped with the promoter GfaABC1D, which allows astrocyte‐specific knockdown of Grin2a. One control shRNA virus pAAV2/9‐GfaABC1D‐EGFP‐3FLAG, and three (1#‐3#) Grin2a‐shRNA viruses (pAAV2/9‐GfaABC1D‐EGFP‐3FLAG‐micro30 shRNA(Grin2a)) with EGFP after expression were produced. AAVs were bilaterally injected into the rat hippocampal dentate gyrus. After 21 days of virus expression and the behavior tests, the rats were sacrificed and the knockdown efficacy of Grin2a was confirmed with immunofluorescent staining (nearly 100%) (Figure 1), the reduced GluN2A at the protein and mRNA levels were also demonstrated (Figure 1).

Next rats were randomly divided into four groups (5–8 rats/group): vehicle group, Aβ group (rats subjected to ICV injection of Aβ_1‐42_ oligomers), shGrin2a group (rats subjected to Grin2a‐shRNA viruses injection), shGrin2a + Aβ group (rats subjected to Grin2a‐shRNA viruses injection before ICV injection of Aβ_1‐42_). After 3 weeks AAVs vectors expression, the rats received an intracerebral ventricular injection of Aβ_1‐42_ oligomers. Three days later, Barnes maze test and New Objective Recognition (NOR) were sequentially conducted (Figure 1a).

Barnes maze test was used to evaluate the spatial learning and memory. After the first day's habituation, the rats were trained from day 2–5. At the third day of the training phase, both Aβ and shGrin2a + Aβ rats showed significantly increased latencies to locate the target box (Figure 1f,g,i), which could not be explained by reduced locomotor activity, as there were no differences among the rats in the average locomotor speed during the training days (Figure 1h). During the probe test of the seventh day, not only Aβ and shGrin2a+Aβ rats, shGrin2a rats also showed significantly increased latencies to enter the target hole, moreover shGrin2a + Aβ rats showed a stronger increase of latency (Figure 1i) (Aβ: 22.33 ± 1.60 s; shGrin2a + Aβ: 77.51 ± 16.27 s versus vehicle: 5.81 ± 1.40 s, *p* < 0.001; shGrin2a: 21.18 ± 4.5 s versus vehicle, *p* < 0.01). These results indicated Aβ exposure or astrocyte‐specific Grin2a knockdown can impair spatial learning and memory, and astrocytic Grin2a knockdown further aggravates Aβ‐induced spatial learning and memory decline.

By the novel object recognition test, we assessed cognition of the rats. The preference index (PI) was used to indicate the rat preference for the novel object (Figure 1j,k). Similar to the vehicle rats, shGrin2a rats spent more time exploring the novel object than that of Aβ or shGrin2a + Aβ rats spent (preference index: Aβ: 0.09 ± 0.04 versus vehicle: 0.29 ± 0.06, *p* < 0.05; shGrin2a + Aβ: 0.01 ± 0.01 versus vehicle, *p* < 0.01; shGrin2a: 0.23 ± 0.07 versus vehicle, *p* > 0.05). What is more, shGrin2a + Aβ rats showed a significant reduction of preference index (preference index: shGrin2a + Aβ: 0.01 ± 0.01 versus Aβ: 0.09 ± 0.04, *p* < 0.05), which indicates astrocyte‐specific Grin2a knockdown deteriorates Aβ‐induced cognitive deficit.

### 2.2 Knockdown of astrocytic Grin2a aggravates Aβ‐induced synaptotoxicity

**FIGURE 2:**
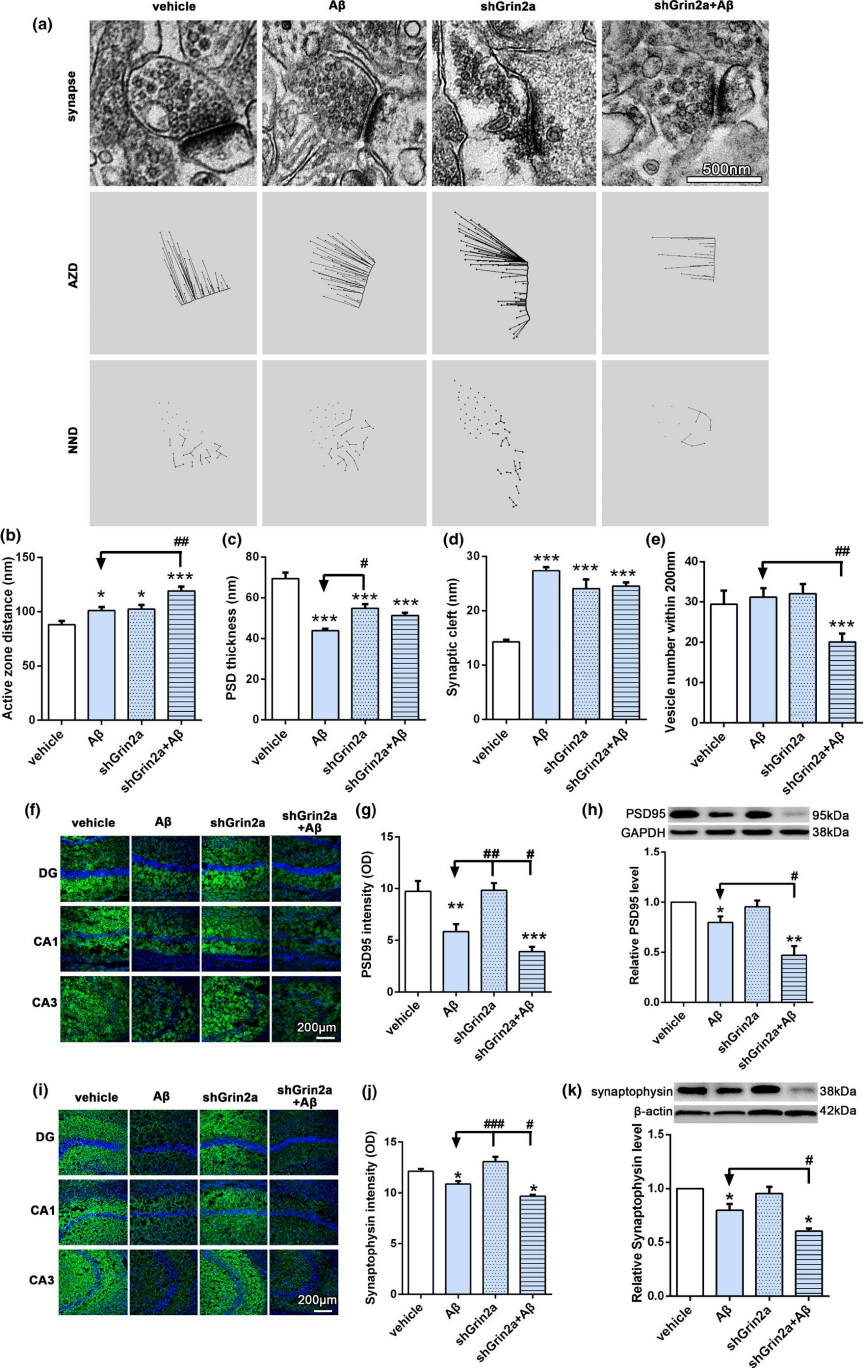
Knockdown of rat astrocytic Grin2a aggravates Aβ‐induced synaptotoxicity. (a–e) Electron microscopy results showed the effects of astrocytic Grin2a knockdown and quantification. *n* = 9–12 synapses from 3 rats/group. AZD, active zone distance; NND, nearest neighbor distance. Bar, 500 nm. (f–h) Representative confocal images (f) and western blot band (h) of PSD95 in the rat hippocampus and quantification (g, h). *n* = 11–16 sections from 3rats/group. Bar, 200 μm. (i–k) Representative confocal images (i) and western blot band (k) of synaptophysin and quantification (j, k). *n* = 8 sections from 3 rats/group. Bar, 200 μm. Numerical data are shown as means ± SEM. * vs. vehicle, *p < 0.05, **p < 0.01, ***p < 0.001, ^#^ vs. Aβ, ^#^ *p* < 0.05, ^##^ *p* < 0.01, ^###^ *p* < 0.001

It is well known that cognitive decline is correlated with synapse degeneration, so we further evaluated the alterations of synapses. As shown in Figure 2a‐e, electron microscopy results showed that Aβ can induce farther vesicle distance from the active zone (Figure 2a,b, Aβ: 101.06 ± 3.32 versus vehicle:88.15 ± 3.32 nm within 200 nm distance of the active zone, *p* < 0.05) and increased width of synapse cleft (Figure 2a,d, Aβ: 27.38 ± 0.70 versus vehicle:14.26 ± 0.43, *p* < 0.001), and a further significant reduction of thickness of PSD (Figure 2a,c, Aβ: 43.77 ± 0.98 versus vehicle: 69.37 ± 2.90, *p* < 0.001), but has no effect on the number of synaptic vesicles with 200nm distance of the active zone. Knockdown of astrocytic Grin2a can further increase Aβ‐induced farther vesicle distance (Figure 2a,b, shGrin2a + Aβ: 119.21 ± 4.0 nm versus Aβ: 101.06 ± 3.32 nm, *p* < 0.001) and reduce the vesicle numbers (Figure 2a,e, shGrin2a + Aβ: 20.09 ± 2.09 versus Aβ: 31.2 ± 2.27, *p* < 0.01), which indicated that astrocyte‐specific Grin2a knockdown can exacerbate the Aβ‐induced deficits of synaptic ultrastructures.

Next, with immunofluorescent staining and western blot methods we evaluated the levels of pre‐ and postsynaptic related proteins PSD‐95 and synaptophysin. Our results showed that Aβ led to the losses of PSD95 and synaptophysin (Figure 2f–h, PSD95 intensity: Aβ: 5.85 ± 0.72 versus vehicle 9.73 ± 1.02, *p* < 0.01; relative level of PSD95: Aβ: 0.76 ± 0.03 versus vehicle, *p* < 0.05; Figure 2i–k, synaptophysin intensity: Aβ: 10.87 ± 0.29 versus vehicle 12.13 ± 0.25, *p* < 0.05; relative level of synaptophysin: Aβ: 0.80 ± 0.06 versus vehicle, *p* < 0.05), while Grin2a knockdown further aggravated Aβ‐induced decreases of PSD95 and synaptophysin (Figure 2f–h, PSD95 intensity: shGrin2a + Aβ: 3.9 ± 0.46 versus Aβ, *p* < 0.05; two‐way ANOVA, *F* = 5.16, *p* < 0.05; relative level of PSD95: shGrin2a + Aβ: 0.47 ± 0.09 versus Aβ, *p* < 0.05; Figure 2i–k, synaptophysin intensity: shGrin2a + Aβ: 9.69 ± 0.14 versus Aβ, *p* < 0.05; relative level of synaptophysin: shGrin2a + Aβ: 0.60 ± 0.02 versus Aβ, *p* < 0.05). In addition, Fluoro‐Jade C staining (Schmued et al., 2005) results showed there was no positive staining neurons (data not shown), which indicating no significant neurodegenerative neurons in the hippocampus. The above data suggest that astrocytic Grin2a knockdown exacerbate Aβ‐induced synaptotoxicity.

### 2.3 Knockdown of astrocytic Grin2a inhibits Aβ‐induced increase of NGF

**FIGURE 3:**
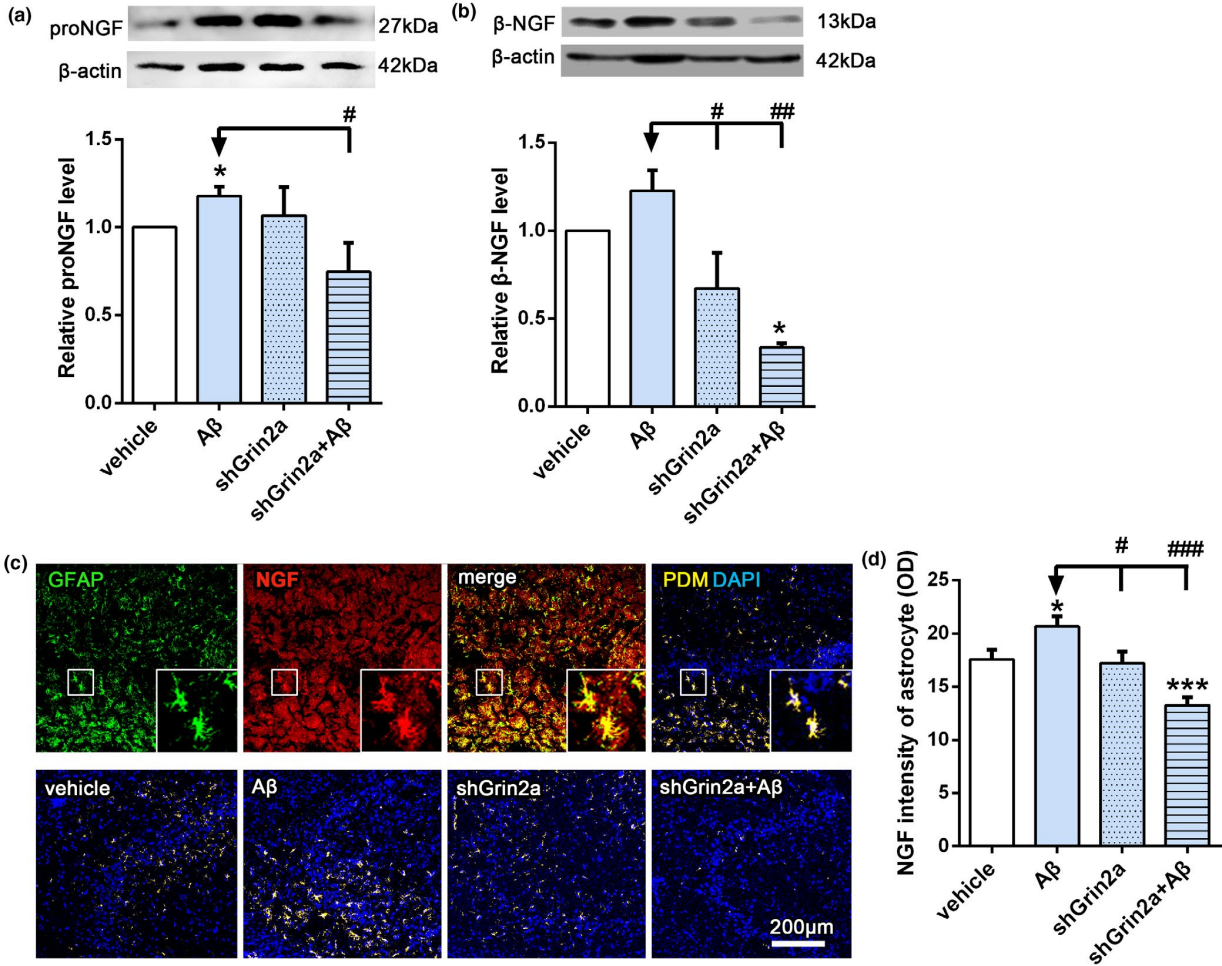
Knockdown of astrocytic Grin2a inhibits the increase of β‐NGF induced by Aβ. (a) Representative western blot band and quantification showing the effects of astrocytic Grin2a knockdown on β‐NGF precursor, proNGF level. (b) Representative western blot band and quantification showing the effects of astrocytic Grin2a knockdown on β‐NGF level. (c) Scheme diagram of confocal images analysis of β‐NGF in astrocytes (GFAP positive) (the upper) and representative quantification in CA3 subregion of the hippocampus (the lower). Bar, 200 μm. d. Quantification of β‐NGF immunofluorescent intensity in the rat hippocampus. *n* = 13–15 sections from 3 rats/group.*vs. vehicle, *p < 0.05, ***p < 0.001, ^#^ vs. Aβ, ^#^ *p* < 0.05, ^##^ *p* < 0.01, ^###^ *p* < 0.001

In light of our previous in vitro findings that astrocytic GluN2A protects synapse against Aβ synaptotoxicity likely through NGF secreted from astrocytes, to further confirm neuroprotective role of GluN2A through regulating NGF in AD rat, we next evaluated the effects of astrocyte‐specific Grin2a knockdown on the level of β‐NGF. As shown in Figure 3, Aβ induced increased β‐NGF expression in the hippocampal astrocytes, characterized with stronger fluorescent intensity of co‐labeled GFAP and β‐NGF, which was in agreement with our previous results, while astrocyte‐specific Grin2a knockdown inhibited Aβ‐induced increase of β‐NGF fluorescent intensity (Figure 3c,d, shGrin2a + Aβ: 13.25 ± 0.74 versus Aβ: 20.66 ± 0.95, *p* < 0.05). Western blot analysis also revealed that Aβ can significantly increase the level of β‐NGF in the hippocampus, astrocytic Grin2a knockdown counteracted Aβ‐upregulated β‐NGF and further reduced it to the level lower than that of the vehicle (Figure 3b, relative level of β‐NGF: shGrin2a + Aβ: 0.33 ± 0.02 versus Aβ: 1.23 ± 0.12, *p* < 0.05; two‐way ANOVA: *F* (1, 8) = 5.8, *p* < 0.05). We also evaluated the level of the precursor of NGF, proNGF. Western blot results showed Aβ induced increase of proNGF, knockdown of astrocytic Grin2a significantly mitigated Aβ‐induced elevation of proNGF level (Figure 3a, relative level of proNGF: shGrin2a + Aβ: 0.75 ± 0.17 versus Aβ: 1.18 ± 0.05, *p* < 0.05; two‐way ANOVA: *F* (1, 18) = 4.46, *p* < 0.05).

### 2.4 Knockdown of astrocytic Grin2a inhibits Aβ‐induced increase of pNF‐κB

**FIGURE 4:**
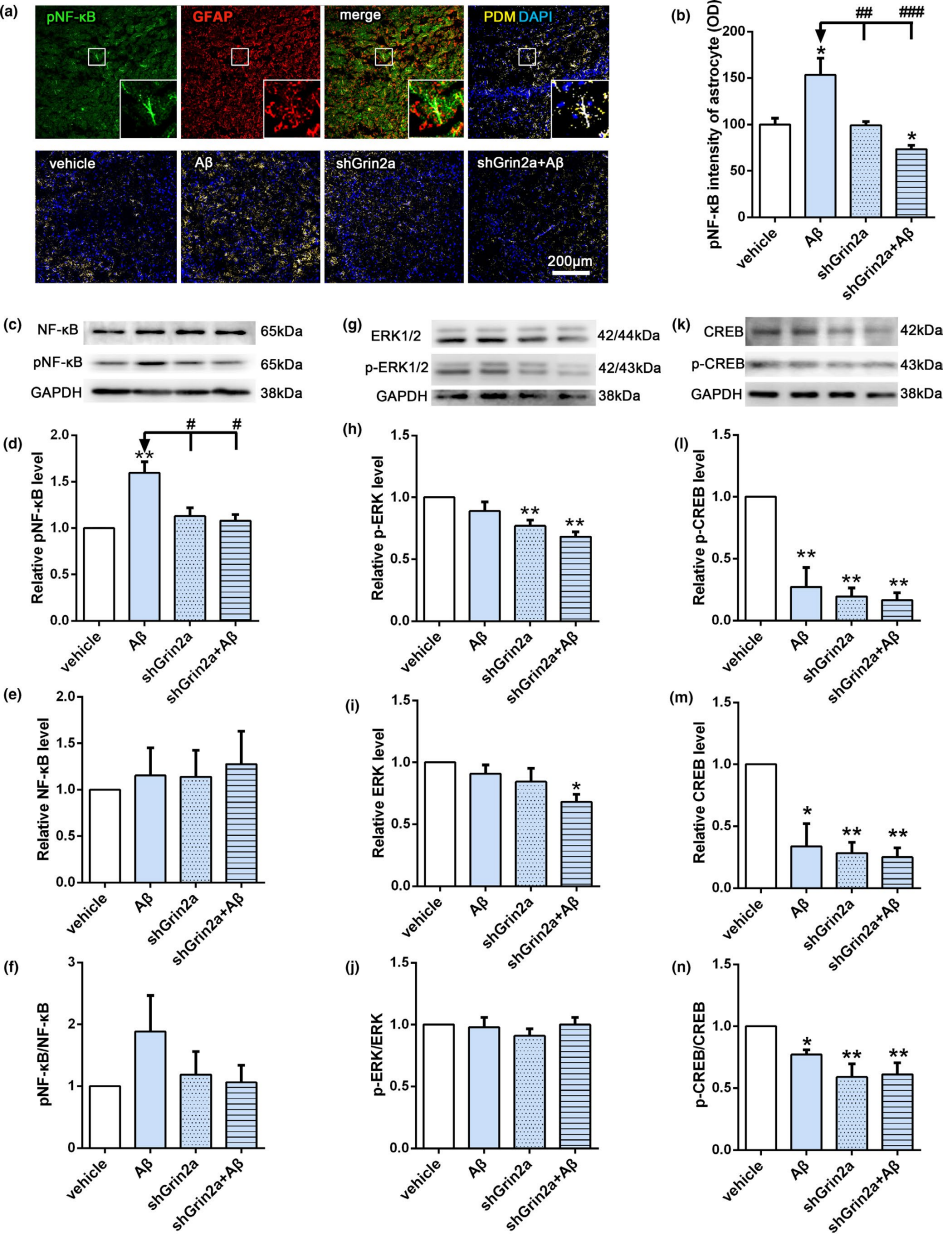
Knockdown of astrocytic Grin2a inhibits the increase of pNF‐κB induced by Aβ. (a, b) Scheme diagram of confocal images analysis of pNF‐κB intensity in astrocytes (GFAP positive) (the upper) and representative quantification in CA3 subregion of the hippocampus (the lower). *n* = 6 sections from 3 rats/group. Bar, 200 μm. (c–n) Representative western blot bands and quantification showing the effects of astrocytic Grin2a knockdown on pNF‐κB, NF‐κB (c–f); pERK, ERK (g–j) and pCREB, CREB (k–n) levels. *n* = 5 rats/group. *vs. vehicle, *p < 0.05, **p < 0.01, ^#^vs. Aβ, ^#^ *p* < 0.05, ^##^ *p* < 0.01, ^###^ *p* < 0.001

To further investigate the mechanism of GluN2A regulating Aβ‐induced increase of astrocytic β‐NGF, we next examined several transcription factors that may affect the synthesis of NGF. As shown in Figure 4a,b, our immunofluorescent results showed that Aβ can elevate pNF‐κB fluorescent intensity in astrocytes, which implying the activation of NF‐κB. While knockdown of astrocytic Grin2a can inhibit Aβ‐induced increased fluorescent intensity of pNF‐κB in astrocytes (Figure 4a,b, shGrin2a + Aβ: 73.49 ± 3.59 versus Aβ: 153.4 ± 18.08, *p* < 0.05). Western blot analysis revealed the similar alterations of pNF‐κB (Figure 4c–f, relative level of pNF‐κB: shGrin2a + Aβ: 1.17 ± 0.06 versus Aβ: 1.84 ± 0.25, *p* < 0.01; two‐way ANOVA, *F* (1, 2) = 14.78, *p* < 0.01). In contrast, astrocytic Grin2a knockdown can reduce the levels of pERK and pCREB (Figure 4g–n), but did not show significant effect on Aβ‐induced decreased pCREB (Figure 4k–n).

### 2.5 Knockdown of astrocytic Grin2a inhibits Aβ‐induced increase of Furin

**FIGURE 5:**
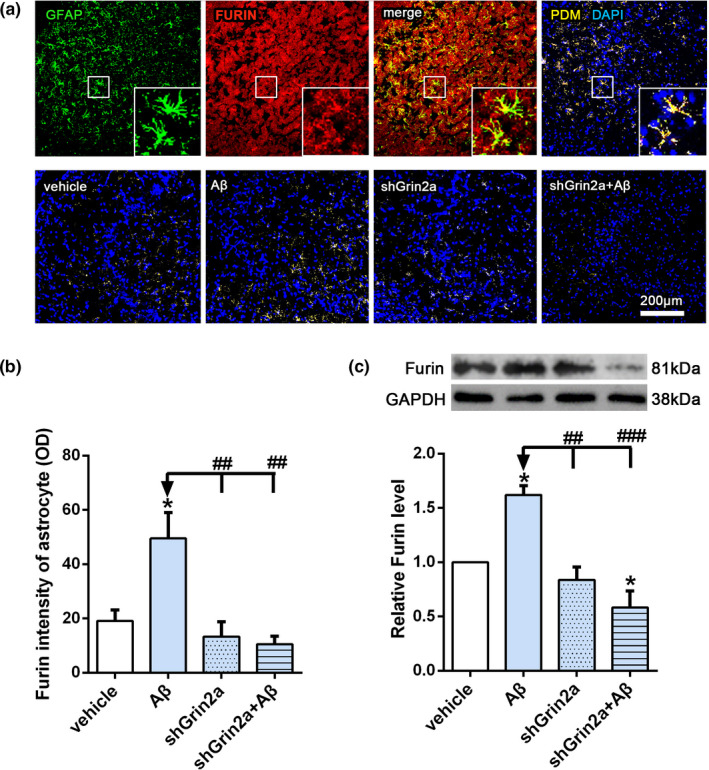
Knockdown of astrocytic Grin2a inhibits Aβ‐induced increase of Furin. (a, b) Scheme diagram of confocal images analysis of Furin intensity in astrocytes (GFAP positive) (the upper) and representative quantification in CA3 subregion of the hippocampus (the lower). Bar, 200 μm. (c–e) Representative western blot bands and quantification of Furin showing the effects of astrocytic Grin2a knockdown on Furin level. *n* = 3 rats/group. *vs. vehicle, *p < 0.05, ^#^vs. Aβ, ^#^ *p* < 0.05, ^##^ *p* < 0.01, ^###^ *p* < 0.001

Furin is an important proprotein convertase which can cleave proNGF and convert it into mature NGF, which is the main form of neuroprotective action. In order to explore whether knockdown of astrocytic Grin2a inhibits Aβ‐induced β‐NGF elevation through regulating NGF maturation, we observed the expression of Furin with immunofluorescence and western blot methods. As shown in Figure 5, Aβ can significantly increase the fluorescence intensity of Furin in astrocytes and total protein level in the hippocampus. However, astrocytic Grin2a knockdown greatly inhibits Aβ‐induced increased fluorescence intensity of Furin in astrocytes (Figure 5a,b, shGrin2a + Aβ: 10.60 ± 2.89 versus Aβ: 49.5 ± 9.51, *p* < 0.05; two‐way ANOVA, *F* = 8.40, *p* < 0.01) and total protein level (Figure 5c, relative level of Furin: shGrin2a + Aβ: 0.58 ± 0.15 versus Aβ: 1.62 ± 0.09, *p* < 0.05; two‐way ANOVA, *F*(1, 8) = 6.88, *p* < 0.001). The results suggest that astrocytic Grin2a knockdown inhibits Aβ‐induced increase of Furin to affect NGF maturation.

### 2.6 Knockdown of astrocytic Grin2a affects Aβ‐induced VAMP3 alterations

**FIGURE 6:**
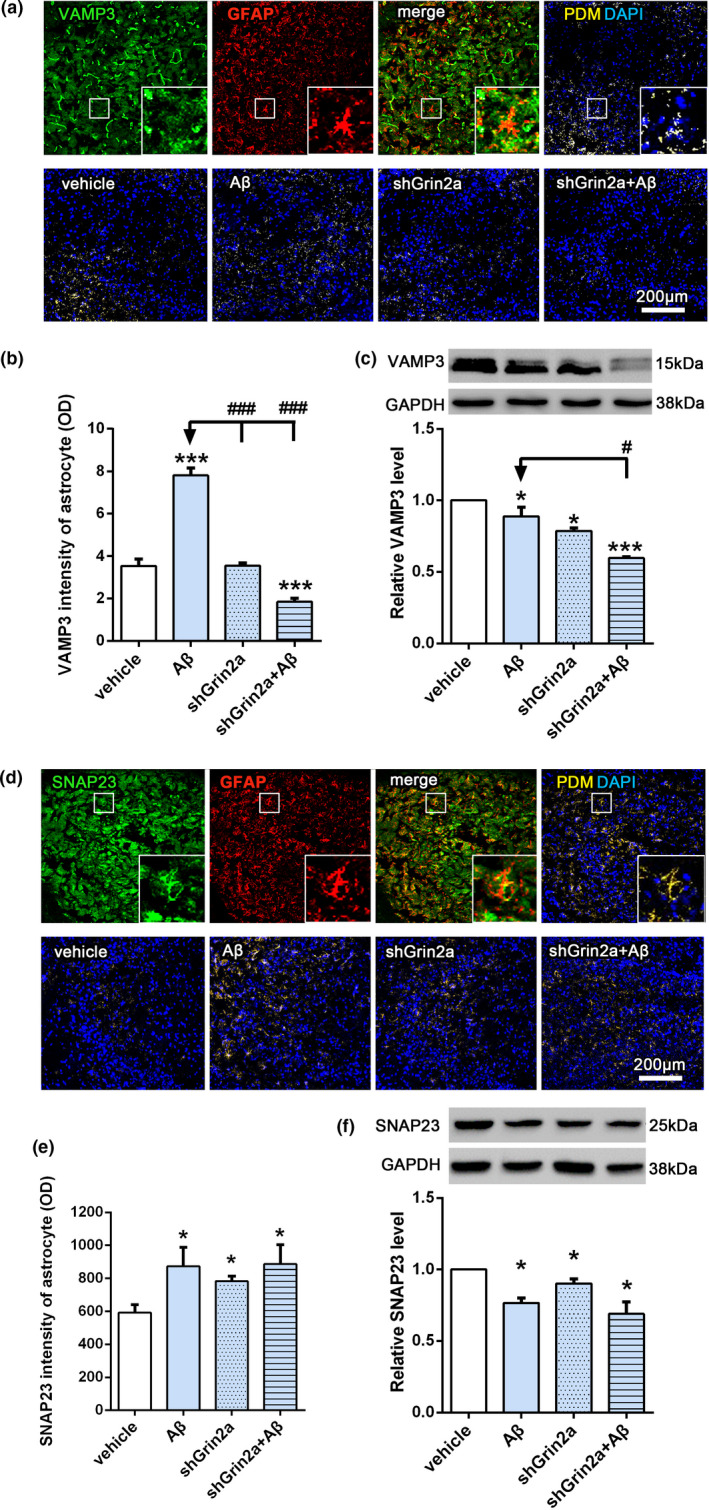
Knockdown of astrocytic Grin2a affects Aβ‐induced VAMP3 alterations. (a, b) Scheme diagram of confocal images analysis of VAMP3 intensity in astrocytes (GFAP positive)(the upper) and representative quantification in CA3 subregion of the hippocampus (the lower). *n* = 10–11 sections from 3 rats/group. Bar, 200 μm. (c) Representative western blot bands and quantification showing the effects of astrocytic Grin2a knockdown on VAMP3 level. *n* = 3 rats/group. (d, e) Scheme diagram of confocal images analysis of SNAP23 intensity in astrocytes (GFAP positive) (the upper) and representative quantification in CA3 subregion of the hippocampus (the lower). *n* = 10–11 sections from 3 rats/group. Bar, 200 μm. (f) Representative western blot bands and quantification showing the effects of astrocytic Grin2a knockdown on SANP23 level. * vs. vehicle, *p < 0.05,**p < 0.01, ^#^ vs. Aβ, ^#^ *p* < 0.05, ^###^ *p* < 0.001

Mature NGF, through vesicle trafficking and exocytosis, can be released into the intercellular space to modulate synaptic activity (Iulita & Cuello, 2014). SNAREs (soluble NSF N‐ethylmaleimide‐sensitive fusion protein attachment protein receptors) are a class of vesicular transport proteins which mediate vesicle trafficking, docking, fusion and cellular exocytosis. SNAREs can be defined as vesicle‐associated SNAREs (v‐SNAREs) or target‐membrane‐associated SNAREs (t‐SNAREs). Vesicle‐associated membrane protein 3 (VAMP3), a member of the v‐SNAREs, plays an important role in vesicle transport. Synaptosomal‐associated protein 23 (SNAP23), belonging to the t‐SNAREs, participates in vesicle transport and fusion. Both of them are highly enriched in astrocytes. To determine whether knockdown of astrocytic Grin2a combined with Aβ injection may affect astrocytic vesicles transport and secretion, we measured the levels of VAMP3 and SNAP23. Our results showed Aβ induced increased fluorescent intensities of VAMP3 (Figure 6a,b) and SNAP23 in astrocytes (Figure 6d,e), while the total protein levels of VAMP3 and SNAP23 decreased significantly (Figure 6c,f). Knockdown of astrocytic Grin2a greatly inhibited Aβ‐induced fluorescent intensities increase of VAMP3 in astrocytes (Figure 6a,b, shGrin2a + Aβ: 1.84 ± 0.18 versus Aβ: 7.8 ± 0.35, *p* < 0.05), and further aggravated Aβ‐induced total protein decrease of VAMP3 (Figure 6c, relative level of VAMP3: shGrin2a + Aβ: 0.60 ± 0.01 versus Aβ: 0.87 ± 0.06, *p* < 0.05), but did not affect Aβ‐induced alterations of SNAP23. The above results suggest knockdown of astrocytic Grin2a affect astrocytic vesicle secretion, maybe finally lead to synaptic impairments.

## 3 DISCUSSION

The data presented here demonstrate that reduction of astrocytic GluN2A in the hippocampus of AD model rat aggravates Aβ oligomer‐induced spatial memory and cognition impairments through regulating astrocytic NGF synthesis, mature and secretion. To the best of our knowledge, our present data are the only in vivo findings published so far through the use of AD rats with a conditional knockdown of astrocytic Grin2a to demonstrate the involvement of the astrocytic NMDA receptor subunit in Aβ‐induced early synaptotoxicity (Skowrońska et al., 2019). Our research shed light on a novel mechanism of astrocytic GluN2A in exerting protective effects on synapses at the early stage of Aβ synaptotoxicity, which could contribute to establish new targets for AD prevention and early therapy.

NGF is an essential mediator of synaptic plasticity. The accumulation of proNGF and an enhanced degradation of the mature NGF has been found in brains of AD patients, which indicated the dysregulation of NGF metabolism (Cuello et al., 2019). NGF addition has been demonstrated to improve cognitive decline in AD (Cuello et al., 2019; Siegel & Chauhan, 2000). Under physiological conditions, NGF is thought to be mainly produced by neurons (Balkowiec & Katz, 2000; Lewin & Barde, 1996; Miklic et al., 2004), while under pathological conditions, astrocytes become the major source of NGF production (Bakhit et al., 1991; Goss et al., 1998). An increased β‐NGF release in astrocytes with Aβ exposure has been described previously by our team and others (Bakhit et al., 1991; Goss et al., 1998; Li et al., 2016; Schulte‐Herbruggen et al., 2007). Our current findings of increased β‐NGF production in AD model rat might reflect an early endogenous autoprotective attempt of astrocytes to minimize Aβ‐induced neuron loss, since there was significant synaptic dysfunction but no obvious neuronal degeneration under our experiment conditions. This view is supported by in vitro AD models and some clinical data available on NGF treatment in early stages of AD (Enciu et al., 2011; Iulita & Cuello, 2014; Schulte‐Herbruggen et al., 2007).

Our results showed that astrocyte‐specific knockdown of Grin2a significantly counteracted Aβ‐induced compensatory elevation of β‐NGF, with concomitant aggravated memory and cognitive decline. Together with our previous study (Li et al., 2016), the present finding further confirmed astrocytic GluN2A could mediate neuroprotection through regulating NGF production in astrocytes. One possible mechanism regulating NGF synthesis by astrocytic GluN2A may be mediated by NF‐κB, since astrocytic Grin2a knockdown inhibited Aβ‐induced activation of NF‐κB. This result is supported by the evidence that sublethal concentrations of Aβ activate NF‐κB through a mechanism involving NMDA receptors (Kawamoto et al., 2008). In addition, a feedback loop between NGF and NF‐κB triggered by Aβ might occur. Through astocytic GluN2A, Aβ activated NF‐κB, in turn increasing NGF that, when interacting with its receptor TrkA, led to further NF‐κB activation and NGF increase (Chacón et al., 2010).

At the same time, under our experimental condition, the involvement of BDNF, another important neurotrophic factor related with synaptic efficacy, could not be excluded. As demonstrated by Miklic et al., (2004), astrocytes have ability to synthesize NGF and BDNF, and Aβ could increase BDNF by activating NF‐κB through NMDA receptor, in which BDNF contributed to mediate a neuroprotective response to Aβ (Kawamoto et al., 2008). Our results showed that either Aβ treatment or knockdown of astrocytic Grin2a caused declined pCREB, which may indicate decreased BDNF production (Tong et al., 2001). Several reports suggest there is a cross talk between NF‐κB and CREB, while BDNF is proposed as a common gene regulated by the two transcription factors (Caviedes et al., 2017; Kaltschmidt & Kaltschmidt, 2015). Whether astrocytic GluN2A can directly or indirectly regulate BDNF under Aβ neurotoxic effect and the precise mechanism need to be clarified in future.

Furin, as a calcium‐dependent serine endoprotease (Yamada et al., 2018), can cleave proNGF and convert it into mature NGF to exert neuroprotective effects. Here our results show that the immunofluorescent intensity of Furin in astrocytes and the total level in hippocampus elevated significantly after Aβ injection, which was consistent with the corresponding change of NGF. Knockdown of astrocytic Grin2a greatly inhibited this elevation. Recently a novel finding implicated furin in NMDA‐induced neuronal injury (Yamada et al., 2018), so we raise the possibility that Aβ‐induced a pronounced increase calcium influx through astrocytic GluN2A resulted in increase of Furin, the latter caused elevated NGF by promoting conversion from proNGF to mature NGF. While knockdown of astrocytic Grin2a led to the reduced Furin level may be one main reason for the decreased β‐NGF and unchanged proNGF level.

After cleaved by Furin, mature NGF can be delivered to the extracellular space through vesicle transport, fusion and exocytosis. During this serial processes, VAMP3 and SNAP23 play important roles (Ravichandran et al., 1996; Schubert et al., 2011). Our results showed that astrocytic Grin2a knockdown strongly inhibited Aβ‐induced increase of VAMP3 in astrocytes and aggaravated the decrease of total VAMP3 level, but did not show any effects on Aβ‐induced alterations of SNAP23. Our finding suggests that astrocytic GluN2A reduction has more critical effects on VAMP3, the sharply decreased VAMP3 disrupted NGF transport and exocytosis, ultimately impaired synaptic efficacy. It has been demonstrated that VAMP3 contributed to Ca^2+^‐dependent trafficking of astrocytic vesicles (Schubert et al., 2011), and the mechanism of astrocytic Grin2a knockdown elicited VMAP3 decrease may be related to astrocytic NMDA receptor‐coupled Ca^2+^ release from the intracellular Ca^2+^ stores (Jimenez‐Blasco et al., 2015; Verkhratsky & Chvátal, 2020). In the meantime, it should be noticed that Aβ and astrocytic Grin2a knockdown led to the decreased total levels of both VAMP3 and SNAP23, so the precise mechanism that how astrocytic GluN2A regulates VAMP3 and SNAP23 in different cells under Aβ exposure remains to be investigated.

There are some limitations to our research. We have examined some key factors associated with NGF synthesis, mature and secretion, but no further assessment for the related regulators necessary for proNGF cleavage and NGF degradation in the extracellular space upon Aβ stimulation. In addition, to sort astrocytes and neurons using fluorescence‐activated cell sorting (FACS) is necessary for neuron‐astrocyte communication research in the future. As a beneficial factor in the early stage of AD, if supplementary NGF to be performed in Grin2a knockdown AD rats to rescue the memory and cognition deficits will make this research more perfect.

**FIGURE 7:**
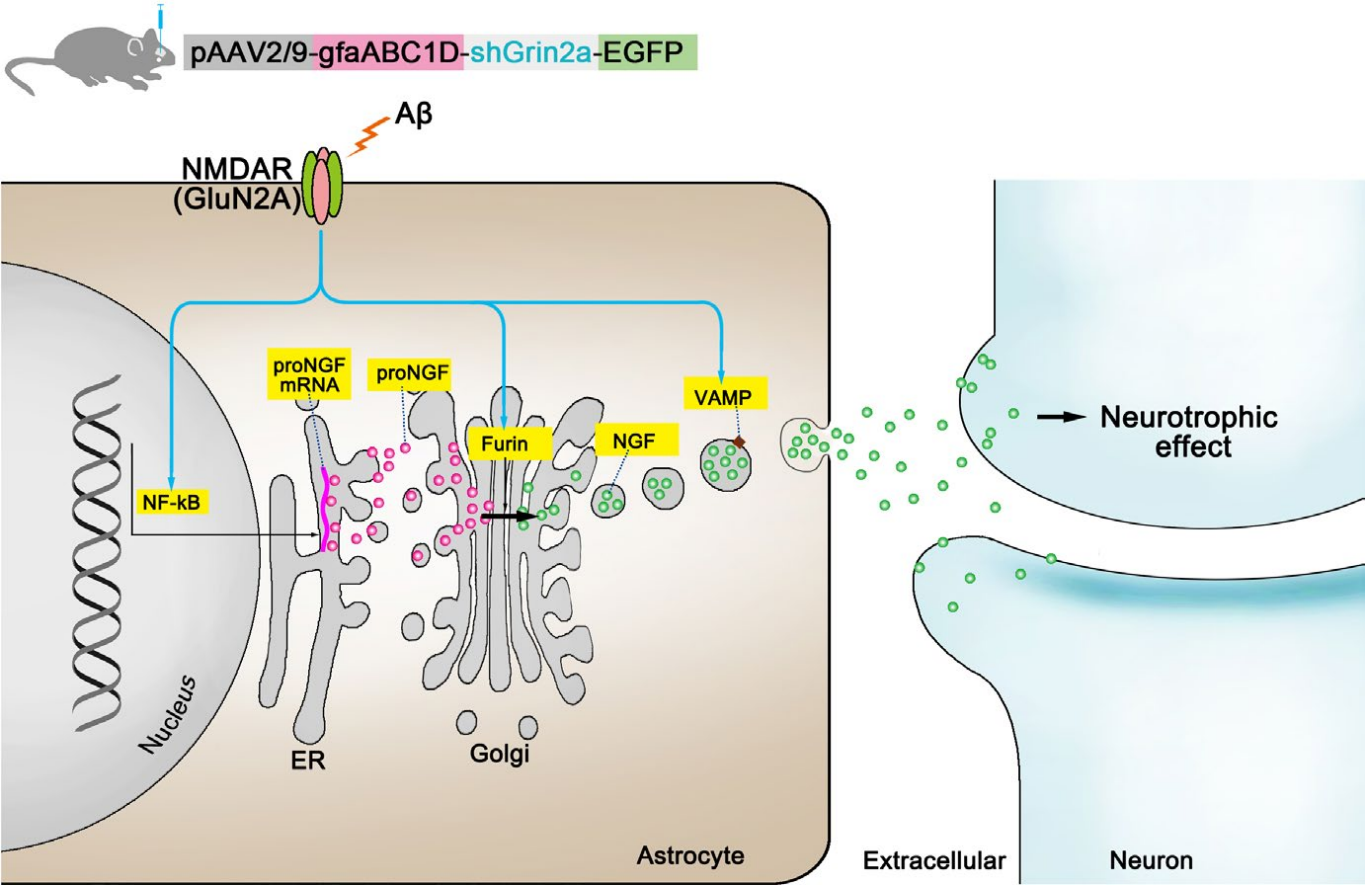
A proposed model for knockdown of astrocytic Grin2a aggravated Aβ‐induced synaptotoxicity. The reduced astrocytic GluN2A can counteract Aβ‐induced compensatory protective increase of β‐NGF through modulating pNF‐κB, Furin and VAMP3, which modulating the synthesis, mature and secretion of NGF respectively

In summary, our present work illustrates the complex nature of neuron‐astrocyte communication. Specifically, our work shows that at the early stage of Aβ oligomers synaptotoxicity, the reduction of astrocytic GluN2A can exacerbate synaptotoxicity through regulating NGF synthesis, mature and secretion (Figure 7). This novel information contributes to establish early targeted interventions to delay development of AD.

## 4 MATERIAL AND METHODS

### 4.1 Animals

All Wistar rats used in this study were obtained from Beijing HFK Bioscience Co., Ltd (Beijing, China). All experimental procedures were approved by the Animal Ethics Committee of Capital Medical University. All possible efforts were made to minimize rats suffering and the number of rats used.

### 4.2 Preparation of Aβ_1–42_ oligomers

Soluble Aβ_1–42_ oligomers were prepared as previously described (Gamarra et al., 2020). Aβ_1–42_ peptides purchased from AnaSpec (Fremont, CA, USA) were dissolved in hexafluoroisopropanol (HFIP, Sigma Aldrich) to 1 mM, aliquoted and dried. The Aβ_1–42_ peptide film was dissolved in dimethylsulfoxide (DMSO) to a concentration of 1mM and then aliquoted and kept at −80℃ until use. The oligomer nature of Aβ_1‐42_ in such preparation was confirmed with atomic force microscopy (AFM) (data not shown).

### 4.3 Virus Generation

The adeno‐associated virus (AAV) shRNA knockdown vector system has become a powerful tool of modulating gene expression. For astrocyte‐specific Grin2a knockdown, GfaABC1D was used as a promoter, AAV vectors were serotyped with AAV2/9 coat proteins (pAAV2/9‐GfaABC1D‐EGFP‐3FLAG‐micro30shRNA(Grin2a) or pAAV2/9‐GfaABC1D‐EGFP‐3FLAG) and produced by OBiO Technology (Shanghai, China) Corp. Ltd. The following short hairpin sequences were used: pAAVE‐1# 5′‐GCACTCCTATGATAACATT‐3′; pAAVE‐2# 5′‐GCAAGTTCTCCCAGGGATA‐3′ and pAAVE‐3# 5′‐GGAGGAGTTTGTGGACCAA‐3′.

### 4.4 Stereotaxic surgery

Adult male Wistar rats (age, 8–10 weeks; weight, 250–300 g) were anesthetized using 3% isofurane, and mounted on a stereotaxic apparatus (Shenzhen RWD Life Science, China). To specifically knockdown Grin2a in the hippocampal astrocytes, AAV (pAAV2/9‐GfaABC1D‐EGFP‐3FLAG‐micro30shRNA(Grin2a) or pAAV2/9‐GfaABC1D‐EGFP‐3FLAG) was stereotaxically injected bilaterally into the hippocampal dentate gyrus (DG) (AP, −3.8 mm from bregma; ML, ±2.4 mm; DV, −3.3 mm). Each rat was injected with 0.5 μL of virus on each side of DG at the rate of 1 μL/min. After 3 weeks of expression of the viral vector, the rats were treated with Aβ_1‐42_ oligomers by a unilateral i.c.v. (AP, −0.8 mm from bregma; ML, ±1.5 mm; DV, −4.5 mm) injection, an equal volume of saline solution was injected in the vehicle rats. Each rat was slowly injected with 10 μL Aβ_1‐42_ (1 μg/μL) in 10 min. The rats were allowed to recover for at least 3 days before behavioral tests. Scheme of entire experimental study is provided in Figure 1a.

### 4.5 Barnes maze test

The Barnes maze test was used for assessing spatial learning and memory. The modified protocol used here was previously described (Sadeghian et al., 2019). A high (90 cm to the floor) black circular platform (120 cm in diameter), containing 20 uniform holes in its periphery (with 19 false boxes and one escape box), was used in the present study. Prior to the first trial, rats were habituated to the escape box and the platform. An acquisition trial consisted of placing a rat in the starting box for 30 s. Then, the box was raised, and an aversive stimulus (bright light) was switched on, and the rat was allowed to explore the maze freely for 240 s. Starting from the second training, the maze was randomly rotated one to several holes, but the target box was always fixed in the same position. The rats were trained 2 times per day for 5 consecutive days. After the sixth day's rest, on the seventh day, the rats were submitted to a probe trial for 240 s on the maze without an escape box. The platform and holes were carefully cleaned with 70% ethanol after every trial. All trials were recorded with an overhead camera and tracked using Smart 3.0 video tracking system (Harvard Apparatus, Holliston, MA, USA). The following behavioral parameters were recorded: the latency of reaching the target box/hole, the average speed and the number of errors, which is defined as the rat's head reaching or exploring any non‐target hole.

### 4.6 Novel object recognition (NOR)

The novel object recognition (NOR) test is based on the nature tendency of rats to show preference for novel objects. The present protocol was previously described (Zappa et al., 2018) with some little modification, three objects A, B and C were prepared, among which A and B are identical, and C are completely different from A and B in shape and color, but with similar dimension. After a 5 min habituation session in an empty arena, the rats were submitted to a training session, in which rats explored two identical objects (A and B) during 5min and the time spent exploring each object was recorded. Objects A and B were placed equidistant from the center and walls of the arena. One hour after the training session, the rats were again placed in the arena for the probe session, one of the two familiar objects (A or B) was replaced with novel object C. Again, the time spent in exploring the familiar object (FO) and novel object (NO) was acquired and analyzed. The location of the novel object was counter‐balanced among animals. In each trial, the rat was allowed to explore the objects freely. Exploration was considered effective when the orientation of the rat's nose was toward the object, within a range of ~1 cm or closer to the object. Climbing was not considered exploration. The objects and the floor were cleaned thoroughly with 70% ethanol between trials. All trials were recorded with Smart 3.0 video tracking system. The preference index (PI) was calculated as the time exploring the novel object divided by total time spent in exploring the familiar and the novel objects in the probe session.

### 4.7 Immunofluorescence and imaging analysis

In brief, brain sections were fixed (ice cold 4% paraformaldehyde), washed, permeabilized (0.3% Triton X‐100 in PBS) and blocked with appropriate non‐immune sera. After that, specimens were incubated (overnight, 4^◦^C) with primary antibodies against mouse anti‐PSD95 (1:500, mouse, MA1‐045, Thermo Fisher), anti‐GFAP (1:500, MAB360; Millipore), rabbit anti‐GFAP (1:5000, Z0334; Dako), anti‐GluN2A (1:500, NBP2‐19551, Novus Biologicals), anti‐furin (1:500, ab183495; abcam), anti‐SNAP23 (1:500, 10825‐1‐AP; proteintech), anti‐VAMP3 (1:500, 10702‐1‐AP; proteintech), anti‐pNF‐κB (1:500, 3033; CST), anti‐synaptophysin (1:500, 36406; CST), the goat anti‐β‐NGF (1:200, AF‐556‐NA; R&D Systems). The secondary antibodies used in the present experiments were Alexa Fluor^®^‐488 or −594 conjugated goat anti‐mouse IgG, and Alexa Fluor^®^‐594 or‐488 conjugated goat anti‐rabbit IgG or donkey anti‐goat Alexa Fluor^®^‐594 conjugated donkey anti‐goat IgG (1:500, Invitrogen). Cell nuclei were visualized with Hoechst 33342 (1:1000) or DAPI. Fluorescence‐labeled optical sections were captured with a Leica confocal laser scanning microscope (TCS SP5). Image J software (W.S. Rasband, U. S. National Institutes of Health, Bethesda, Maryland, USA) was used for immunofluorescent intensity quantitative analysis of brain sections.

### 4.8 Western blotting

In brief, total cellular proteins of the rats hippocampi were extracted, homogenized and subsequent brief sonication. Homogenates were centrifuged at 4℃ (30 min, 14000 rpm), the pellet was discarded and protein contents of lysates were determined using the BCA protein assay (Thermo). Total protein from each sample was boiled for 5 min in loading buffer (1% SDS, 1 M DTT, 0.5 M Tris HCl pH 6.8, 20% glycerol and 2% bromoxylenol blue), separated on 8–12% polyacrylamide gel (SDS‐PAGE), and transferred onto PVDF membranes. Membranes were then blocked with 5% skim milk in Tris buffered saline tween 20 (TBST) buffer before overnight incubation at 4℃ with the following primary antibodies: mouse anti‐PSD95 (1:1000, MA1‐045, Thermo Fisher), anti‐GAPDH (1:1000, sc‐32233; Santa Cruz), β‐actin (1:3000, SAB1305554, Sigma); rabbit anti‐GluN2A (1:1000, NBP2‐19551, Novus Biologicals), anti‐furin (1:1000, ab183495; Abcam), anti‐SNAP23 (1:1000, 10825‐1‐AP; proteintech), anti‐VAMP3 (1:1000, 10702‐1‐AP; Proteintech), anti‐p‐NF‐κB (1:1000, 3033; CST), anti‐p‐CREB (1:1000, 9198; CST), anti‐p‐ERK (1:1000, 4370; CST), anti‐synaptophysin (1:1000, 36406; CST), anti‐proNGF (1:1000, ANT‐005, Alomone labs); goat anti‐β‐NGF (1:2000, AF‐556‐NA; R&D Systems), followed by incubation with goat anti‐mouse or anti‐rabbit IgG (H+L)‐HRP, or rabbit anti‐goat IgG (H+L)‐HRP (1:5000, Absin) in TBST. Protein bands were acquired using ECL Plus, and analyzed in a luminescent image analyzer system (LAS‐500, GE Healthcare). Densitometric quantification was carried out using Image J software. The results were expressed as the band densities of the target protein/β‐actin or GAPDH ratio and then normalized to the values from the corresponding controls.

### 4.9 Transmission electron microscopy and synapse morphometric analysis

The rats were deeply anesthetized and perfused transcardially with PBS for 1 min, then with a fixative composed of 0.25% glutaraldehyde and 4% paraformaldehyde in 0.1 M PB (pH 7.4). The hippocampus was isolated and cut into ~1 mm^3^ cubes, post‐fixed in 1% osmium tetroxide, dehydrated, embedded in Epon resin 812, before ultrathin sections (70 nm) were prepared and collected on copper grids. Sections were stained with 2% uranyl acetate/lead citrate, and grids were examined with a HITACHI H‐7700 electron microscope (acceleration voltage: 100 kV). Thickness of postsynaptic density (PSD) was evaluated with Image J software, and the number of the vesicles and the vesicle‐active zone distance (within 200 nm distance of active zones) were measured with LoClust software (Nikonenko & Skibo, 2004).

### 4.10 Quantitative RT‐PCR

Total RNA from the rat hippocampus was extracted using an RNeasy Plus mini kit (Qiagen, Hilden, Germany). cDNA was synthesized using an QuantiTect Reverse Transcription Kit (Qiagen, Hilden, Germany). Quantitative PCR was performed on a Bio‐Rad CFX96 thermal cycler using SsoAdvanced™ Universal SYBR^®^ Green Supermix (Bio‐Rad, Hercules, CA, USA). The primer sequences for Grin2a were 5′‐TCAGCATTGTCACCTTGGAG‐3′ (sense) and 5′‐CTTCACATTCATCCCTTCGTTG‐3′ (antisense). Cycling parameters were 30 s at 95℃ followed by 40 cycles for 10 s at 95℃, 30 s at 60℃. Target gene expression was normalized to the levels of the internal standard β‐actin.

### 4.11 Statistical analysis

Data are depicted as mean ± standard error (SE). Statistical analysis (SPSS 13.0 or Prism 8.0 software) included one‐way ANOVA or *T*‐test for comparison between two groups, two‐way ANOVA for interaction of two factors, *p* < 0.05 was considered significant.

## CONFLICT OF INTEREST

The authors declare no conflict.

## AUTHOR CONTRIBUTIONS

Y.W. and L.R.C conceived and designed the project. Z.S.D, X.Y.C., W.N.Z. performed the experiments. H.L, G.T.Z and Y.Z.S. analyzed the data and drafted the manuscript.

## Data Availability

The data that support the findings of this study are available from the corresponding author upon reasonable request.

## References

[acel13437-bib-0001] Bai, N., Aida, T., Yanagisawa, M., Katou, S., Sakimura, K., Mishina, M., & Tanaka, K. (2013). NMDA receptor subunits have different roles in NMDA‐induced neurotoxicity in the retina. Molecular Brain, 6, 34. 10.1186/1756-6606-6-34 23902942 PMC3733768

[acel13437-bib-0002] Bakhit, C., Armanini, M., Bennett, G. L., Wong, W. L., Hansen, S. E., & Taylor, R. (1991). Increase in glia‐derived nerve growth factor following destruction of hippocampal neurons. Brain Research, 560, 76–83. 10.1016/0006-8993(91)91217-o 1836973

[acel13437-bib-0003] Balkowiec, A., & Katz, D. M. (2000). Activity‐dependent release of endogenous brain‐derived neurotrophic factor form primary sensory neurons detected by ELISA in situ. Journal of Neuroscience, 20, 7417–7423. 10.1523/JNEUROSCI.20-19-07417.2000 11007900 PMC6772775

[acel13437-bib-0004] Cahoy, J. D., Emery, B., Kaushal, A., Foo, L. C., Zamanian, J. L., Christopherson, K. S., Xing, Y., Lubischer, J. L., Krieg, P. A., Krupenko, S. A., Thompson, W. J., & Barres, B. A. (2008). A transcriptome database for astrocytes, neurons, and oligodendrocytes: A new resource for understanding brain development and function. Journal of Neuroscience, 28, 264–278. 10.1523/JNEUROSCI.4178-07.2008 18171944 PMC6671143

[acel13437-bib-0005] Canu, N., Amadoro, G., Triaca, V., Latina, V., Sposato, V., Corsetti, V., Severini, C., Ciotti, M. T., & Calissano, P. (2017). The Intersection of NGF/TrkA signaling and amyloid precursor protein processing in Alzheimer's disease neuropathology. International Journal of Molecular Sciences, 18, 1319. 10.3390/ijms18061319 PMC5486140 28632177

[acel13437-bib-0006] Caviedes, A., Lafourcade, C., Soto, C., & Wyneken, U. (2017). BDNF/NF‐kappaB signaling in the neurobiology of depression. Current Pharmaceutical Design. 23, 3154–3163. 10.2174/1381612823666170111141915 28078988

[acel13437-bib-0007] Chacón, P. J., Arévalo, M. A., & Tébar, A. R. (2010). NGF‐activated protein tyrosine phosphatase 1B mediates the phosphorylation and degradation of I‐kappa‐Balpha coupled to NF‐kappa‐B activation, thereby controlling dendrite morphology. Molecular and Cellular Neuroscience. 43, 384–393. 10.1016/j.mcn.2010.01.005 20123020

[acel13437-bib-0008] Chang, L., Zhang, Y., Liu, J., Song, Y., Lv, A., Li, Y., Zhou, W., Yan, Z., Almeida, O. F., & Wu, Y. (2016). Differential regulation of N‐Methyl.D‐aspartate receptor subunits is an early event in the actions of soluble amyloid β (1–40) oligomers on hippocampal neurons. Journal of Alzheimer's Disease, 51, 197–212. 10.3233/JAD-150942 26836185

[acel13437-bib-0009] Conti, F., Barbaresi, P., Melone, M., & Ducati, A. (1999). Neuronal and glial localization of NR1 and NR2A/B subunits of the NMDA receptor in the human cerebral cortex. Cerebral Cortex, 9, 110–120. 10.1093/cercor/9.2.110 10220224

[acel13437-bib-0010] Conti, F., DeBiasi, S., Minelli, A., & Melone, M. (1996). Expression of NR1 and NR2A/B subunits of the NMDA receptor in cortical astrocytes. Glia, 17, 254–258. 10.1002/(SICI)1098-1136(199607)17:3<254:AID-GLIA7>3.0.CO;2-0 8840166

[acel13437-bib-0011] Cuello, A. C., Pentz, R., & Hall, H. (2019). The brain NGF metabolic pathway in health and in Alzheimer's pathology. Frontiers in Neuroscience, 13, 62. 10.3389/fnins.2019.00062 30809111 PMC6379336

[acel13437-bib-0012] Dumas, T. C. (2005). Developmental regulation of cognitive abilities: Modified composition of a molecular switch turns on associative learning. Progress in Neurobiology, 76, 189–211. 10.1016/j.pneurobio.2005.08.002 16181726

[acel13437-bib-0013] Enciu, A. M., Nicolescu, M. I., Manole, C. G., Mureşanu, D. F., Popescu, L. M., & Popescu, B. O. (2011). Neuroregeneration in neurodegenerative disorders. BMC Neurology, 11, 75. 10.1186/1471-2377-11-75 21699711 PMC3146817

[acel13437-bib-0014] Ferreira, I. L., Ferreiro, E., Schmidt, J., Cardoso, J. M., Pereira, C. M., Carvalho, A. L., Oliveira, C. R., & Rego, A. C. (2015). Aβ and NMDAR activation cause mitochondrial dysfunction involving ER calcium release. Neurobiology of Aging, 36, 680–692. 10.1016/j.neurobiolaging.2014.09.006 25442114

[acel13437-bib-0015] Gamarra, M., Blanco‐Urrejola, M., Batista, A. F. R., Imaz, J., & Baleriola, J. (2020). Object‐based analyses in FIJI/ImageJ to measure local RNA translation sites in neurites in response to Aβ1‐42 oligomers. Frontiers in Neuroscience, 14, 547. 10.3389/fnins.2020.00547 32581689 PMC7284234

[acel13437-bib-0016] Goss, J. R., O'Malley, M. E., Zou, L., Styren, S. D., Kochanek, P. M., & DeKosky, S. T. (1998). Astrocytes are the major source of nerve growth factor upregulation following traumatic brain injury in the rat. Experimental Neurology, 149, 301–309. 10.1006/exnr.1997.6712 9500953

[acel13437-bib-0017] Guo, Z. H., & Mattson, M. P. (2000). Neurotrophic factors protect cortical synaptic terminals against amyloid and oxidative stress‐induced impairment of glucose transport, glutamate transport and mitochondrial function. Cerebral Cortex, 10, 50–57. 10.1093/cercor/10.1.50 10639395

[acel13437-bib-0018] Iulita, M. F., & Cuello, A. C. (2014). Nerve growth factor metabolic dysfunction in Alzheimer's disease and Down syndrome. Trends in Pharmacological Sciences, 35, 338–348. 10.1016/j.tips.2014.04.010 24962069

[acel13437-bib-0019] Jimenez‐Blasco, D., Santofimia‐Castaño, P., Gonzalez, A., Almeida, A., & Bolaños, J. P. (2015). Astrocyte NMDA receptors’ activity sustains neuronal survival through a Cdk5‐Nrf2 pathway. Cell Death & Differentiation, 22, 1877–1889. 10.1038/cdd.2015.49 25909891 PMC4648333

[acel13437-bib-0020] Kaltschmidt, B., & Kaltschmidt, C. (2015). NF‐KappaB in long‐term memory and structural plasticity in the adult mammalian brain. Frontiers in Molecular Neuroscience, 8, 1–11. 10.3389/fnmol.2015.00069 26635522 PMC4656838

[acel13437-bib-0021] Kawamoto, E. M., Lepsch, L. B., Boaventura, M. F., Munhoz, C. D., Lima, L. S., Yshii, L. M., Avellar, M. C., Curi, R., Mattson, M. P., & Scavone, C. (2008). Amyloid beta‐peptide activates nuclear factor‐kappaB through an N‐methyl‐D‐aspartate signaling pathway in cultured cerebellar cells. Journal of Neuroscience Research, 86, 845–860. 10.1002/jnr.21548 17969100

[acel13437-bib-0022] Lee, M. C., Ting, K. K., Adams, S., Brew, B. J., Chung, R., & Guillemin, G. J. (2010). Characterisation of the expression of NMDA receptors in human astrocytes. PLoS One, 5, e14123. 10.1371/journal.pone.0014123 21152063 PMC2994931

[acel13437-bib-0023] Lewin, G. R., & Barde, Y. A. (1996). Physiology of the neurotrophins. Annual Review of Neuroscience, 19, 289–317. 10.1146/annurev.ne.19.030196.001445 8833445

[acel13437-bib-0024] Li, Y., Chang, L., Song, Y., Gao, X., Roselli, F., Liu, J., Zhou, W., Fang, Y., Ling, W., Li, H., Almeida, O. F., & Wu, Y. (2016). Astrocytic GluN2A and GluN2B oppose the synaptotoxic effects of amyloid‐β1‐40 in hippocampal cells. Journal of Alzheimer's Disease, 54, 135–148. 10.3233/JAD-160297 27497478

[acel13437-bib-0025] Liu, J., Chang, L., Roselli, F., Almeida, O. F., Gao, X., Wang, X., Yew, D. T., & Wu, Y. (2010). Amyloid‐β induces caspase‐dependent loss of PSD‐95 and synaptophysin through NMDA receptors. Journal of Alzheimer's Disease, 22, 541–556. 10.3233/JAD-2010-100948 20847396

[acel13437-bib-0026] Mattson, M. P. (2008). Glutamate and neurotrophic factors in neuronal plasticity and disease. Annals of the New York Academy of Sciences, 1144, 97–112. 10.1196/annals.1418.005 19076369 PMC2614307

[acel13437-bib-0027] Miklic, S., Juric, D. M., & Carman‐Krzan, M. (2004). Differences in the regulation of BDNF and NGF synthesis in cultured neonatal rat astrocytes. International Journal of Developmental Neuroscience, 22, 119–130. 10.1016/j.ijdevneu.2004.03.001 15140465

[acel13437-bib-0028] Müller, U. C., Deller, T., & Korte, M. (2017). Not just amyloid: physiological functions of the amyloid precursor protein family. Nature Reviews Neuroscience, 18, 281–298. 10.1038/nrn.2017.29 28360418

[acel13437-bib-0029] Myers, S. J., Yuan, H., Kang, J. Q., Tan, F. C. K., Traynelis, S. F., & Low, C. M. (2019). Distinct roles of GRIN2A and GRIN2B variants in neurological conditions. F1000Research, 8, 1940. 10.12688/f1000research.18949.1 PMC6871362 31807283

[acel13437-bib-0030] Nikonenko, A. G., & Skibo, G. G. (2004). Technique to quantify local clustering of synaptic vesicles using single section data. Microscopy Research and Technique, 65, 287–291. 10.1002/jemt.20134 15662622

[acel13437-bib-0031] Ravichandran, V., Chawla, A., & Roche, P. A. (1996). Identifification of a novel syntaxin‐ and synaptobrevin/VAMP‐binding protein, SNAP‐23, expressed in non‐neuronal tissues. Journal of Biological Chemistry, 271, 13300–13303. 10.1074/jbc.271.23.13300 8663154

[acel13437-bib-0032] Sadeghian, A., Fathollahi, Y., Javan, M., Shojaei, A., Kosarmadar, N., & Rezaei, M. (2019). Spatial learning and memory in barnes maze test and synaptic potentiation in Schaffer collateral‐CA1 synapses of dorsal hippocampus in freely moving. Basic and Clinical Neuroscience, 10, 461–468. 10.32598/bcn.9.10.330 32284835 PMC7149949

[acel13437-bib-0033] Schmued, L. C., Stowers, C. C., Scallet, A. C., & Xu, L. (2005). Fluoro‐Jade C results in ultra high resolution and contrast labeling of degenerating neurons. Brain Research, 1035, 24–31. 10.1016/j.brainres.2004.11.054 15713273

[acel13437-bib-0034] Schubert, V., Bouvier, D., & Volterra, A. (2011). SNARE protein expression in synaptic terminals and astrocytes in the adult hippocampus: A comparative analysis. Glia, 59, 1472–1488. 10.1002/glia.21190 21656854

[acel13437-bib-0035] Schulte‐Herbruggen, O., Hamker, U., Meske, V., Danker‐Hopfe, H., Ohm, T. G., & Hellweg, R. (2007). Beta/A4‐Amyloid increases nerve growth factor production in rat primary hippocampal astrocyte cultures. International Journal of Developmental Neuroscience, 25, 387–390. 10.1016/j.ijdevneu.2007.05.010 17646078

[acel13437-bib-0036] Selkoe, D. J. (2002). Alzheimer's disease is a synaptic failure. Science (New York, NY), 298, 789–791. 10.1126/science.1074069 12399581

[acel13437-bib-0037] Shooter, E. M. (2001). Early days of the nerve growth factor proteins. Annual Review of Neuroscience, 24, 601–629. 10.1146/annurev.neuro.24.1.601 11283322

[acel13437-bib-0038] Siegel, G. J., & Chauhan, N. B. (2000). Neurotrophic factors in Alzheimer's and Parkinson's disease brain. Brain Research. Brain Research Reviews, 33, 199–227. 10.1016/s0165-0173(00)00030-8 11011066

[acel13437-bib-0039] Skowrońska, K., Obara‐Michlewska, M., Zielińska, M., & Albrecht, J. (2019). NMDA receptors in astrocytes: in search for roles in neurotransmission and astrocytic homeostasis. International Journal of Molecular Sciences, 20, 309. 10.3390/ijms20020309 PMC6358855 30646531

[acel13437-bib-0040] Tong, L., Thornton, P. L., Balazs, R., & Cotman, C. W. (2001). b‐Amyloid1–42 impairs activity‐dependent cAMP‐response element‐binding protein signaling in neurons at concentrations in which cell survival is not compromised. Journal of Biological Chemistry, 276, 17301–17306. 10.1074/jbc.M010450200 11278679

[acel13437-bib-0041] Verkhratsky, A., & Chvátal, A. (2020). NMDA receptors in astrocytes. Neurochemical Research, 45, 122–133. 10.1007/s11064-019-02750-3 30767094

[acel13437-bib-0042] Wang, Y., & Mandelkow, E. (2016). Tau in physiology and pathology. Nature Reviews Neuroscience, 17, 5–21. 10.1038/nrn.2015.1 26631930

[acel13437-bib-0043] Wang, Z. C., Zhao, J., & Li, S. (2013). Dysregulation of synaptic and extrasynaptic N‐methyl‐D‐aspartate receptors induced by amyloid‐beta. Neuroscience Bulletin, 29, 752–760. 10.1007/s12264-013-1383-2 24136243 PMC5562550

[acel13437-bib-0044] Yamada, M., Hayashi, H., Yuuki, M., Matsushima, N., Yuan, B., & Takagi, N. (2018). Furin inhibitor protects against neuronal cell death induced by activated NMDA receptors. Scientific Reports, 8, 5212. 10.1038/s41598-018-23567-0 29581474 PMC5980093

[acel13437-bib-0045] Young, M., Blanchard, M. H., Sessions, F., & Boyle, M. D. (1988). Subunit structure of high molecular weight mouse nerve growth factor. Biochemistry, 27, 6675–6681. 10.1021/bi00418a005 3143401

[acel13437-bib-0046] Zappa Villar, M. F., López Hanotte, J., Falomir Lockhart, E., Trípodi, L. S., Morel, G. R., & Reggiani, P. C. (2018). Intracerebroventricular streptozotocin induces impaired Barnes maze spatial memory and reduces astrocyte branching in the CA1 and CA3 hippocampal regions. Journal of Neural Transmission, 125, 1787–1803. 10.1007/s00702-018-1928-7 30244292

